# Understanding the low uptake of long-acting reversible contraception by young women in Australia: a qualitative study

**DOI:** 10.1186/s12905-015-0227-9

**Published:** 2015-09-10

**Authors:** Cameryn C. Garrett, Louise A. Keogh, Anne Kavanagh, Jane Tomnay, Jane S. Hocking

**Affiliations:** Gender and Women’s Health Unit, Centre for Health Equity, Melbourne School of Population and Global Health, The University of Melbourne, Carlton, Victoria Australia; Centre for Excellence in Rural Sexual Health, Rural Health Academic Centre, The University of Melbourne, Shepparton, Victoria Australia; Centre for Epidemiology and Biostatistics, Melbourne School of Population and Global Health, The University of Melbourne, Carlton, Victoria Australia

## Abstract

**Background:**

Australia has high rates of teenage pregnancy compared with many Western countries. Long-acting reversible contraception (LARC) offers an effective method to help decrease unintended pregnancies; however, current uptake remains low. The aim of this study was to investigate barriers to LARC use by young women in Australia.

**Methods:**

Healthcare professionals were recruited through publicly available sources and snowball sampling to complete an interview about young women’s access to and use of LARC. The sample consisted of general practitioners, nurses, medical directors of reproductive and sexual health organisations, a sexual health educator, and health advocates. In addition, four focus groups about LARC were conducted with young women (aged 17–25 years) recruited via health organisations and a university. The data were analysed thematically.

**Results:**

Fifteen healthcare professionals were interviewed and four focus groups were conducted with 27 young women. Shared barriers identified included norms, misconceptions, bodily consequences, and LARC access issues. An additional barrier identified by young women was a perceived lack of control over hormones entering the body from LARC devices. Healthcare professionals also raised as a barrier limited confidence and support in LARC insertions. Strategies identified to increase contraceptive knowledge and access included increasing nurses’ role in contraceptive provision and education, improving sex education in schools, and educating parents.

**Conclusions:**

Challenges remain for young women to be able to make informed choices about contraception and easily access services. More research is needed around innovative approaches to increase LARC knowledge and access, including examining the role of nurses in enhancing young women’s reproductive health.

## Background

Poor access to contraception and contraceptive failure due to poor adherence contributes to high unintended pregnancy and abortion rates among young women in Australia compared with many Western countries [1]. Within Australia, the number of abortions among women aged under 30 years is more than double the number for women aged 30 years or above [2]. Unintended pregnancies can have significant social, psychological, physical and economic costs, particularly for young women. Teenage mothers have higher rates of anxiety and depression, are more likely to live in disadvantage and are at higher risk for social exclusion than women who become pregnant at an older age [3, 4]. Even decades after adolescents give birth, they are more likely to experience mental health disorders, have lower levels of education, workforce participation, and income than women who give birth at an older age [5].

Long-acting reversible contraception ([LARC] i.e. contraceptive implant and intrauterine devices), which do not require daily adherence, offer an effective method to help decrease unintended pregnancies. Studies such as the Contraceptive CHOICE Project in the United States have demonstrated high efficacy, acceptability and continuation rates of LARC. In the Contraceptive CHOICE Project, women were provided with standardised contraceptive counselling and offered the contraceptive method of their choice free of charge [6]. Women in the study who used LARC were 21 times less likely to become pregnant than women using short-acting methods like oral contraceptives [7]. Of the 1,400 participants aged 14–19 years, over 70 % chose a LARC [6]. At 12 and 24 months, LARC users had higher levels of satisfaction and continuation rates than oral contraceptive users [8, 9].

Despite the potential of LARC to decrease rates of unintended pregnancies, the current use of LARC in Australia remains very low compared with many European countries [10]. Oral contraceptives are the most popular form of contraception in Australia; 48 % of women using contraception are using oral contraceptives, compared with only 5 % using an IUD and 5 % using an implant [11]. In addition, a recent survey of general practice activities reported that only 15.4 per 100 contraceptive consultations are for prescribing LARC compared with 68.6 per 100 contraceptive consultations for the combined oral contraceptive pill in women aged 12–54 years [12]. There is, however, some evidence of higher uptake of LARC among women living in non-metropolitan areas [13]. International research has highlighted a number of barriers to LARC use including healthcare providers’ limited or out-of-date information about LARC and patients’ limited awareness of and misinformation about LARC e.g. [14, 15]. Less is known about the barriers to LARC use in the Australian context. Given the particularly low uptake of LARC in Australia [10], more research is needed to understand the barriers to LARC use in this context in order to enhance LARC uptake.

The aim of this study was to investigate the barriers to young women’s use of LARC in Australia and to identify possible approaches for increasing LARC knowledge and access. This included exploring the potential role primary healthcare nurses could play in increasing LARC uptake in the general practice (primary care) setting. Currently contraception counselling and provision is largely provided by clinicians. In order to gain a broad, contextualised understanding of LARC attitudes in Australia, we aimed to utilise both lay expertise and professional expertise through examining the views of both young women and healthcare professionals.

## Methods

Focus groups and semi-structured interviews were used to obtain in-depth information about young women’s and healthcare professionals’ views of the most effective forms of LARC (the contraceptive implant, the progestogen-only intrauterine device [IUD], and the copper IUD). The contraceptive implant and IUDs have efficacy rates of over 99 % with both typical and perfect use. As little is known about young women’s and healthcare professionals’ perspectives on LARC in Australia, a qualitative methodology was deemed most appropriate for this study.

### Setting

Most contraception provisions Australia take in place in general practice clinics where general practitioners (primary care physicians) provide over 80 % of Australia’s primary care [16]. The general practitioner prescribes the contraception and then the patient is usually required to purchase the contraception from a pharmacy. Patient fees for the general practitioner consultation are subsided either in full or in part by Medicare (Australia’s universal healthcare scheme) depending on the price the general practice charges for the service [17], and the cost of the contraception is subsidised by the Pharmaceutical Benefits Scheme, the Australian Government [18]. In addition to general practice, there are about 20 specialist family planning clinics located in metropolitan towns across the country that also provide contraception and can insert and remove LARC.

Over 60 % of general practice clinics employ at least one practice nurse who undertakes a number of preventative care activities on behalf of the general practitioner including Pap smears and chlamydia tests. While internationally, in countries such as the United Kingdom, nurses play a larger role in contraceptive provision [19], nurses are not currently undertaking these tasks on a large-scale in Australia. Primary healthcare nurses, if appropriately trained, could decrease general practitioner workload and address unmet patient needs by providing LARC education and insertion of the contraceptive implant.

### Interviews with healthcare professionals

In order to obtain a diverse, yet informed range of perspectives, purposive sampling was used. Healthcare professionals interested in young women’s reproductive health were identified through publicly available sources, such as clinic websites, and snowball sampling. We identified the following groups as those most closely involved in the provision of LARC to young women: general practitioners (primary care physicians), nurses with professional experience and knowledge around young women’s reproductive health, directors of sexual and reproductive health services, sexual health educators and health advocates. Individuals working in each of these professions were invited to take part in a semi-structured interview about LARC access and utilisation among young women in Australia. Potential participants were sent an invitation letter, a participant information sheet and a consent form and were asked to post the signed consent form back to the researchers using the reply-paid envelope provided. Written informed consent was obtained from all participants. In the interview, healthcare providers were asked to draw on their clinical experience with young women at their current organisation, as well as experiences with young women in any previous reproductive health role. Healthcare advocates (comprising one advocate in the disability field and one advocate working with migrant and refugee youth) were asked to draw on their professional experience with young women’s reproductive health in their current and previous roles. Ten of the interviews were conducted with healthcare professionals who worked in a major city; five worked regionally. Interviewees were also asked about existing or possible approaches for increasing contraceptive knowledge and access, including increasing primary healthcare nurses’ role in contraceptive services.

Data collection occurred between January and June 2013. Data saturation was reached after the 13th interview. We conducted data analysis concurrent with data collection and assessed saturation using the code for barriers to LARC use. By interview 13, no new barriers were identified during the interview, therefore new recruitment was ceased. No new barriers were identified during interview 14 and 15. The interviews were conducted either by telephone or in-person, depending on the participant’s preference. Interviews ranged in length from 30 min to 1.5 h.

### Focus groups with young women

Women were purposefully recruited from metropolitan and regional areas of Victoria, Australia’s second most populous state. Regional young women were recruited through a community health service and metropolitan young women were recruited through online advertisements at women’s health organisations and a university to take part in a focus group discussion about contraceptive choices. Women recruited through the community health service were informed that their decision about participation would not impact on their access to services. To be eligible women needed to be aged 16–25 years and be sexually active. In accordance with the National Health and Medical Research Council’s National Statement on Ethical Conduct in Human Research (2007), only 16 and 17 year olds who were deemed to be mature minors were invited to participate in the study. During recruitment, the young person was verbally informed about the aims of the study, what participation in the study involved, and participant confidentiality. Sixteen and 17 year olds were then asked to state back to the recruiter their understanding about what participating in the study will involve, what giving their consent means, and about confidentiality in regards to the study. The recruiter then only invited the young person to participate if she could determine that the young person was mature enough to fully understand the research project and was able to give informed consent to participate in the research project. Before the focus group, women were emailed a participant information sheet and consent form. Written informed consent was received on the day of the focus group.

The regional focus groups occurred in a town of 80,000 people located 150 km from Melbourne, the capital city in the state of Victoria. Metropolitan focus groups were held in Melbourne, which has a population of 4 million in the metropolitan region. At the start of the focus group, women filled out a short demographic survey. To maintain confidentiality, women were asked to use a pseudonym in the focus group. During the focus group, after women were asked about their initial knowledge of and attitudes towards LARC, women were given detailed information about LARC including the options available, advantages and any adverse effects. Women were asked about: their contraceptive knowledge, awareness and attitudes towards LARC, factors influencing one’s decision to use or not use, different types of contraception, and approaches to increase contraceptive access and knowledge. In addition to the digital recording, hand-written notes were taken in each focus group. Data collection occurred between September and October 2013. Data saturation was assessed using the code for barriers to LARC use, and given a new barrier was identified during the final focus group, saturation may not have been reached. Each focus group lasted around one hour and participants were compensated for their time with a $30 gift card.

### Analysis

All interviews and focus groups were digitally recorded. The data were transcribed verbatim and analysed thematically [20]. The data from healthcare professionals were analysed first. Transcripts were read and re-read and then coded manually. Next, codes were organised and grouped together into initial themes/patterns emerging from the data, with repeated reference to the transcripts. Themes and sub-themes were further refined and organised through the development of thematic maps and a hierarchy of themes was developed. All transcripts were then re-read in light of the identified themes and to discern thematic evidence in each transcript. The young women’s focus group transcripts were then read and re-read in light of the healthcare professionals’ coding framework. The data from young women were compared and contrasted to the data from healthcare professionals, taking careful note of differences and commonalities in themes between the responses of the young women and healthcare professionals. The coding framework was then further refined with repeated reference to all transcripts. The hierarchy of themes was devised through detailed discussions between CG and LK. Any disagreements in coding were resolved through discussions with all authors and the final thematic framework was agreed upon by all authors.

The study received ethics approval from the University of Melbourne Human Ethics Committee (#1238813 and #1239003).

## Results

### Characteristics of participants

#### Key informants

The 15 healthcare professionals from metropolitan (*n =* 10) and regional locations (*n =* 5) comprised of general practitioners (*n =* 3), nurses (*n =* 7), medical directors of reproductive and sexual health organisations (*n =* 2), a sexual health educator in schools (*n =* 1), and health advocates (*n =* 2). All but one was female and all but one resided in the state of Victoria.

#### Focus groups

Four focus groups with a total of 27 young women were conducted. Participants ranged in age from 17–25 years. Seventeen of the participants were born in Australia; 10 were born overseas. Three women were currently using LARC, 17 were using a non-LARC method and 7 were not using any contraception. Two of the 27 young women were mothers. A total of four women had either completed secondary school or were still at school and 23 were either still at university or had completed their university education. The regional and metropolitan focus groups differed demographically. Metropolitan participants tended to be older, were more likely to be born overseas, had higher education levels, and were less likely to have used LARC than regional participants. These differences may be explained in part due to different recruitment approaches; metropolitan women were recruited mainly from a university, thus their older age, higher levels of education, and more international composition.

### Barriers to LARC uptake

Our analysis identified several barriers to LARC access and uptake reported by the healthcare professionals and young women. These main themes have been divided into shared barriers to LARC use raised by both healthcare professional and young women and barriers raised only by healthcare professionals or young women. Quotations to support these barriers are detailed in Table 1, with further discussion below.

**Table 1:** Barriers to LARC use among young women

Shared barriers reported by young women and healthcare professionals		Illustrative quotations from young women	Illustrative quotations from healthcare professionals
Norms	Receiving a script for oral contraceptives from general practitioners the norm	“I think alotta younger kids also don’t know too much about the rest of the contraceptives, so they just go to the doctor and say, ‘I want the pill’. That’s what they get.” (Regional focus group 2)	“If someone goes along to their GP (general practitioner) for a script for the pill, the GP probably just gives them a script for the pill. I don’t know whether they always necessarily assess whether that’s the best contraception for them and how they are going with it and whether they’re aware or interested in other methods.” (Participant 4, medical director, major city)
	Not the norm for young people to use IUDs	“[The IUD is] not common [in our age group] … I’m sure my mom had this, yes, and my neighbours, yes. This is a common method for their age, I think 30 to 40.” (Metropolitan focus group 2)	“Most young people just rule an IUD out.” (Participant 5, medical director, major city)
Bodily consequences	LARC changes bleeding patterns	“I know some people might wanna be able to determine when they want their period and when they don’t. With the Implanon, it affects people differently. Some people might get their period lots and some don’t get it all.” (Regional focus group 2)	“We are unable to predict how somebody’s going to react to [Implanon] in terms of bleeding … unfortunately with the Implanon they are taking a calculated risk.” (Participant 9, nurse, regional)
	LARC implanted in the body semi-permanently	“The thing about the pill is that there’s not as much of a commitment as some of the other [types of contraception] like the IUD and Implanon. If they don’t work, it could be you have to get it removed.” (Metropolitan focus group 2)	“Well we heard reports … people who feel that if someone feels [the contraceptive implant] in their arm, and they’re in a social setting, that they will be considered to be sluts … I’ve heard women reporting back of that happening in pubs where men will come and it’s pretty bad. Rub your arms, you know, so they don’t want the device sitting there.” (Participant 8, nurse, major city)
Misconceptions	Misconceptions about IUDs	“I’ve talked with my doctor. I’ve heard they try not to give [IUDs] to younger women.” (Metropolitan focus group 2)	“I think really mostly the education GPs have is not to give Mirena to young people. Unless they’ve had children.” (Participant 15, general practitioner, regional)
	Misconception that condoms and pills are the only option	“I think with the long term contraception stuff, it’s about people being more aware that there’s not just the pill. I think, you’re right, people always said, until your early 20s, you just sort of think, ‘Oh, it’s just the pill. That’s my only option’. Because it’s not talked about in schools. No one ever talked about Implanon or anything in school.” (Metropolitan focus group 1)	“A lot of people just don’t have the knowledge or awareness of the LARC methods. I’ve had women that say, ‘Oh, look, I tried the pill and I tried lots of different sorts and they didn’t work for me, so there’s nothing else I can do.’ I mean they’re not even aware of other methods.” (Participant 4, medical director, major city)
LARC access issues	Cost, time and distance	Participant: “Cost [is an obstacle], because your parents might not know [you are using contraception].”	“Recently we had a young girl who … wanted the copper IUD. It took us … probably three or four months for her to actually in the end have that inserted … the pharmacy locally actually didn’t have [the IUD] on stock so they had to order them in from [a nearby town] … By the time she’d had her specialist appointment, picked up her copper IUD and then had it inserted … It ended up costing her about $500.” (Participant 9, nurse, regional)
		Participant: “Yeah [your parents] might not know so you might not be able to have the money to afford to buy a script.” …	
		Participant: “If your parents don’t know you’ve gotta catch buses and stuff like that, and sometimes that can be a bit hard with appointment times.” (Regional focus group 2)	
Barrier reported by young women only			
Perceived lack of control over hormones entering the body	“I don’t like the idea that you’re not in control with [the contraceptive implant]. With the pill, you take it. If that thing stops up, how do you know? You can’t.” (Metropolitan focus group 2)		
	“The rod in the arm … Just sounds really unappealing. Yeah, the whole idea of control and there’s just this thing that’s pumping out hormones, but you can’t stop it if it goes outta hand.” (Metropolitan focus group 2)		
Barrier reported by healthcare professionals only			
Limited confidence and support in LARC insertions	“You need to be quite confident to do [IUD insertions]. Particularly in general practice, where you maybe feel somewhat alone with it and not very supported … You need to get enough volume through to keep your skill up”. (Participant 6, general practitioner, major city)		
	“If you don’t do [Implanon insertions] often enough then it becomes nerve wracking just suddenly if you haven’t put one in for six months or something to suddenly have to put one in. It’s like everyone, everyone has the same feelings, you know. You’d get nervous about it.” (Participant 12, nurse/midwife, major city)		

#### Shared barriers

The shared barriers voiced by both young women and healthcare professionals  related to norms, bodily consequences, misconceptions and LARC access issues.

##### Norms

*Receiving a script for oral contraceptives from general practitioners the norm*. For most women, oral contraceptives were one of the first forms of contraception used and, along with condoms, were the most popular type of contraception among friends. The popularity and familiarity of oral contraceptives in turn propagated a continuing cycle of women using oral contraception. Young women and healthcare professionals noted that it was common for women to ask a doctor specifically for “the pill” when wanting some form of contraception because it was the only contraceptive method they were familiar with. Young women expressed frustration that healthcare providers in these cases did not inform them about alternative contraceptive methods and just assumed that women had knowledge of all methods. Without receiving education about the different types of contraception, many women had little awareness that LARC was even an option.

*Not the norm for young people to use IUDs*. While the contraceptive implant was gaining some popularity, the IUD remained a device clouded in mystery for many young women. Some women in the focus groups had never heard of an IUD and many knew little more than the name. This was confirmed by healthcare professionals who reported that in their professional experience, IUDs were not an option many young women would even consider; rather the IUD was viewed as a method for older women.

##### Bodily consequences

*LARC changes bleeding patterns.* The potential for irregular bleeding was a major deterrent to LARC usage, making women feel like they had little control over their own body. This irregular bleeding was a significant risk that women considered and impeded many women from switching to LARC. The potential for uncontrolled irregular bleeding was in direct contrast to women’s experience on oral contraceptives because they gave women the power to regulate their own menstruation and skip a withdrawal bleed whenever desired. Menstruation was also a tool women used to self-monitor their fertility, with each withdrawal bleed confirming that they were not pregnant. The potential for LARC to result in amenorrhea disconnected women from their bodies and required them to rely on technology like pregnancy tests to confirm the efficacy of the contraception. Women expressed concern that if menstruation stopped completely they would not be able to tell if they had fallen pregnant or if the medication had simply altered their bleeding pattern.

*LARC implanted in body semi-permanently.* The LARC device being placed in the body semi-permanently was also a barrier to LARC use. Not only did this mean a potentially invasive and painful insertion, but that one could not opt-out of the method without assistance from a healthcare provider. Thus, the ultimate control over women’s reproductive health was placed in the hands of others. This was compared with oral contraceptives that women could stop using without medical intervention.

The bodily location of the device was perceived as also potentially hindering LARC use. Some women were uncomfortable about the possibility of feeling the implant in their arm. The implant could also be an unwanted marker of sexual activity. Two healthcare professionals recounted stories of women being labelled as “sluts” after men had felt the implant in the arm. While the IUD was more hidden, it did still require an examination, which was an uncomfortable prospect to some women. Healthcare professionals also reported that the need for a vaginal examination was a deterrent to many young women.

##### Misconceptions

*Misconceptions about IUDs.* Mothers and healthcare providers were implicated in frequently passing on misinformation about IUDs. As a result of some doctors’ out-dated knowledge of IUDs, IUDs were often not discussed in consultations with young women. Both healthcare professionals and young women told stories of doctors advising women against using IUDs. The propagation of out-dated information about the safety of IUDs in nulliparous young women meant that IUDs were not even presented as a potential method to many women in contraceptive consultations.

*Misconception that condoms and pills are the only option.* The interviews and focus groups highlighted that many young women were unable to make informed choices about contraception due to both a limited knowledge about which contraceptive methods were available and a limited understanding of how these methods worked. Many women in the focus groups were not familiar with IUDs, and some were also unaware of the contraceptive implant. Limited awareness of LARC was attributed by young women and healthcare professionals mainly to the fact that in contraceptive consultations in general practice and in sex education classes at school, LARC was rarely, if ever, mentioned. As a result, some women had perceived the oral contraceptives and condoms as the only options available.

##### LARC access issues (cost, time and distance)

Healthcare professionals raised a range of access barriers including long wait times for LARC appointments, high up-front costs (due in part to the limited number of doctors who did not charge patients out-of-pocket for LARC insertion), and the distance travelled, particularly for rural patients, to a healthcare provider that offered LARC services. It was not uncommon for women to wait months before an IUD was inserted. Women typically had to visit the pharmacy to physically pick up the LARC device before it was inserted, which could be a barrier for young women without good access to transportation. Rural youth in particular highlight cost barriers and transport difficulties with trying to match up bus timetables with appointments, especially if one did not want their parents to know that they were visiting a clinic to access contraception.

#### Barrier reported by young women only

##### Perceived lack of control over hormones entering the body

In one metropolitan focus group, multiple women discussed a lack of trust in LARC methods. Despite being informed about LARC’s high efficacy, they expressed limited confidence in the delivery of hormones from LARC devices. With oral contraceptives women knew exactly when and what quantity of hormones was entering their body as they actively swallowed a pill. By contrast, with the IUD or implant, they were unable to regulate the amount of hormones being released from a device inside their body and were concerned that the device might either stop releasing hormones or rather pump out large quantities of hormones. To them, there was a perceived degree of uncertainty and limited control with LARC compared with oral contraceptives.

#### Barrier reported by healthcare professionals only

##### Limited confidence and support in LARC insertion

Regional healthcare professionals reported difficulty accessing LARC training nearby and follow-up support practicing insertions following training. Low uptake rates of IUDs and the contraceptive implant meant that some metropolitan and regional healthcare providers did not have enough clinical experience to maintain competency inserting LARC, and that it was not uncommon for doctors to, for example, receive IUD training, but then never perform an insertion in their practice. Without demand to maintain skills, and with a limited number of other trained inserters on staff, healthcare providers may not be confident to perform insertions. This may require the woman to instead visit a specialist, which would increase wait times, travel, and costs.

### Strategies to increase contraceptive knowledge and access

Multiple strategies were recommended to increase contraceptive knowledge and access. These included increasing young people’s LARC knowledge by discussing LARC in sex education in schools and healthcare providers informing patients about LARC during contraceptive consultations. Young women suggested that LARC education could occur through contraception information nights at health centres, and through credible, fun and interactive websites on contraception. Healthcare professionals stressed the importance of educating young women and men not just in schools, but also in alternative settings, including outreach programs and using peer education. It was noted that contraceptive educational materials needed to be made more inclusive and easier to access through using simple and easy-to-understand wording, providing information that was culturally appropriate and available in different languages, and also providing information in multiple formats including audio and video. Other strategies included general practice clinics advertising that LARC insertions and removals were available at their clinic, and increasing the number of clinics that provided insertions at no cost to the patient, as currently LARC insertions could cost hundreds of dollars for women visiting a specialist.

Young women and healthcare professionals highlighted the need not only to educate young people, but parents as well as many parents were ill-informed about the methods themselves and could spread misinformation about the devices. Young women shared that if parents were more educated about the methods, they would be more likely to discuss LARC with their children. Additionally, education was needed to ensure healthcare providers were up-to-date with the safety and appropriateness of providing young nulliparous women with IUDs. Young women also wanted healthcare professionals to be proactive in informing women about the different types of contraceptive methods available.

#### *Increasing primary healthcare nurses' role in contraceptive counselling and provision*

Healthcare professionals were asked their views on enhancing LARC access through increasing primary healthcare nurses’ role in contraceptive counselling and the provision of the contraceptive implant. The vast majority were supportive of this approach, noting that it was a simple procedure, that nurses often have more time than doctors for an in-depth contraceptive consultation, and that increasing the role of nurses in this way would help decrease wait times for insertions of the contraceptive implant and increase access (Table 2). However, barriers were also discussed, including ensuring that the clinic’s insurance covered nurses for such procedures, time constraints, the scope of nurses’ role, limited awareness that nurses were allowed to insert the contraceptive implant, and that contraceptive provision may be perceived as overstepping into the general practitioner’s sphere, potentially resulting in resistance from general practitioners. An additional barrier noted was that in the current Medicare scheme (Australia’s national publicly funded medical insurance scheme), only services provided by doctors are reimbursed. This might limit the financial viability of the service being provided by nurses. When asked in the focus groups their views on increasing nurses’ role in contraceptive counselling and provision, young women were supportive of this approach, noting that they were happy to speak to either a doctor or a nurse, but many preferred a female healthcare provider.

**Table 2:** Illustrative quotations on increasing the role of primary healthcare nurses in contraceptive services

• “I think as nurses we can have more time to sit and talk and explain the process. It’s the same with the Pap tests. It’s just a sensible thing to have nurses doing [contraceptive implant insertions], and it’s a very mechanical—it’s not a difficult process.” (Participant 14, nurse, regional)
• “[If nurses inserted the contraceptive implant] that would be awesome … It would increase access for the young people so much … We’d be able to meet the demand of the Implanons that young people want to get put in … There’s a lot of practice nurses as well now who are Pap test providers and they might see [doing Implanon insertions] as adding a little bit extra. Therefore just being able to be a little bit more comprehensive when they’re doing a sexual and reproductive health consult.” (Participant 9, nurse, regional)
• “I think of all the procedures that there are in general practice, that would be one of the easier ones that a practice nurse could do … I’m sure you’d get GPs who stomp their feet about it and say, ‘No! No! That’s not right! They should be kept down in the dark ages where they were’. I think you are just gonna get your old-school doctor resistance.” (Participant 6, general practitioner, major city)
• “I don’t know. I would have to think about that, whether that would be the way to go or not. Whether it is a nurse’s responsibility to insert contraception … I don’t know where that would sit within the scope of practice for nurses.” (Participant 11, nurse, regional)

## Discussion

There was high concurrence between young women and healthcare professionals on the barriers to LARC usage. These barriers included norms, bodily consequences, misconceptions, and LARC access issues. Healthcare professionals also highlighted healthcare providers’ limited confidence and support in LARC insertions as an obstacle. An additional barrier reported by young women was the perceived lack of control over hormones entering their body when using a LARC device. This point was not made by healthcare providers, as they are likely to have more confidence in the devices and the delivery of hormones. It is important to understand women’s lack of faith in the hormonal delivery by these devices and how to address this in future education and health promotion. Overall, these findings are consistent with international literature that has reported that women’s limited knowledge of LARC, the familiarity and normality of oral contraceptives, the potential role of healthcare providers in limiting access to these methods and the invasive nature of the devices are major barriers to LARC usage [15, 21–23].

There are a number of limitations that must be considered when interpreting the results of our study. Firstly, we cannot be confident that further themes would not be identified from young women if we continued data collection. This is because young women are a diverse and heterogeneous group, and despite a sample size of 27 and the identification of five shared and well characterised barriers, further, less common, barriers may exist. Secondly, we purposively targeted healthcare professionals with an interest in sexual and reproductive health so that we could gain an in-depth understanding of the barriers to LARC uptake from well-informed professionals. To ensure diverse views were represented, we recruited healthcare professionals from different sectors including general practice, community health, management and policy and sexual and reproductive health education and training. So while we are not able to generalise to the whole population of healthcare professionals treating young women, we are confident that we have captured the views of key stakeholders interested in the sexual and reproductive health of young women. These key informants self-selected to participate in this study, so it is possible that that those who chose to take part had a more favourable view of IUDs and the contraceptive implant than healthcare professionals who did not chose to take part. Thirdly, in this qualitative study, we did not have a large enough sample of each group of health professionals to systematically compare differences in themes between the different groups of healthcare professionals. However, it was reassuring that data saturation was reached; as it indicates that the participants all stressed similar barriers. The richness of our results comes from being able to compare and contrast the viewpoints of two different groups to determine which barriers and facilitators are shared or contrasted between these two critical groups. Finally, while the efficacy of the contraceptive implant and IUD are similar, some barriers to LARC uptake are method specific and some factors may be a larger barrier for one method than another. For example, the requirement of a vaginal examination was a barrier only for the IUD. However, this barrier did fall under the higher theme of “bodily location of the device” which was perceived as a barrier for both the IUD and contraceptive implant.

It is concerning that over a decade after the introduction of the contraceptive implant and the progesterone-only intrauterine device in Australia, many of the young women knew little about these methods. More needs to be done to ensure that young women are aware and knowledgeable about the contraceptive methods available to them. For young women to make informed choices about contraception, they need access to quality contraceptive information and counselling. From the young women’s and healthcare professionals’ accounts, LARC was rarely, if ever, discussed in sex education in schools or in consultations in general practice. Given the reliance in Australia on general practice for contraceptive advice and access, this must change if we wish to see a reduction in unintended pregnancy in this younger age group. Parents could also play an important role in improving young women’s knowledge of contraceptive options. Better education is needed not only for young people, but also for the healthcare professionals who were reported both in our study, and in previous research, as at times acting as gatekeepers, limiting women’s knowledge and access to LARC [24].

Structural barriers must also be addressed to increase access to LARC. As many women access contraception through general practice, practitioners in this setting need to be trained and confident in LARC insertions. This could be achieved, in part, by increasing access to training programs (particularly in rural areas) and opportunities for practitioners to gain experience in insertions through, for example, practitioners partaking in a day of supervised insertions in setting such as family planning clinics that have higher numbers of patients requesting LARC. The up-front cost of LARC could potentially be reduced by lowering the cost of the device, increasing the number of general practice clinics that insert and remove LARC devices at no cost to the patient (bulk-billing clinics), and by increasing the Medicare rebate clinicians receive for inserting and removing LARC. Costs may be particularly high for rural youth with less access to bulk-billing clinics, high transportation costs, and pharmacies that may charge higher prices due to limited competition [25, 26].

We were interested in examining participants’ views on improving contraceptive knowledge and access through increasing the role of primary healthcare nurses around contraceptive counselling and provision. General practice is usually the first point of call for young adults seeking advice on contraception [27] because of the limited number of family planning and sexual health clinics in Australia. However, during a consultation with a general practitioner, the ability to provide contraceptive counselling is limited by time pressures on doctors, making it less likely that patients receive comprehensive counselling about all their contraceptive options. Our data demonstrated support for expanding primary healthcare nurses’ role around contraception from both healthcare professionals and young women. Utilising primary healthcare nurses could increase access to LARC services, particularly in rural areas.

## Conclusions

This study has identified some of the barriers and potential facilitators to LARC uptake in Australia. The results highlight the challenges that remain for young women to be informed about the full range of contraception choices and for easy access to LARC and stress the importance of directing funding and resources to developing approaches to improve young women’s (and men’s) contraceptive knowledge and access. Positive steps could include making discussions on LARC compulsory in sex education in schools and re-educating healthcare providers about the importance of discussing all contraceptive options in medical consultations. These changes may enhance women’s knowledge about LARC, and thereby increase their faith in the efficiency and safety of the devices. Additionally, more research is needed to further explore the potential role of primary healthcare nurses in enhancing young adults’ reproductive health.

## Abbreviations

GP: General practitioner
LARC: Long-acting reversible contraception
IUD: Intrauterine device
